# Silent myocardial infarction fatty scars detected by coronary calcium score CT scan in diabetic patients without history of coronary heart disease

**DOI:** 10.1007/s00330-023-09940-2

**Published:** 2023-08-02

**Authors:** Sara Boccalini, Marie Teulade, Emilie Paquet, Salim Si-Mohamed, Fabio Rapallo, Caroline Moreau-Triby, Sybil Charrière, Nathan Mewton, Loic Boussel, Cyrille Bergerot, Philippe Douek, Philippe Moulin

**Affiliations:** 1 Department of Cardiovascular Radiology, Hôpital Pradel, Hospices Civils de Lyon, Lyon, France; 2 University Claude Bernard Lyon 1, Lyon, France; 3 Department of Endocrinology Louis Pradel University Hospital, Hospices Civils de Lyon, INSERM UMR 1060, Carmen, Lyon, France; 4 Department of Nuclear Medicine, Hôpital Louis Pradel, Hospices Civils de Lyon, Lyon, France; 5 Department of Economics, University of Genova, Genoa, Italy; 6 Department of Cardiology, Hôpital Louis Pradel, Hospices Civils de Lyon, Lyon, France; 7 Department of Radiology, Hôpital de la Croix Rousse, Hospices Civils de Lyon, Lyon, France

**Keywords:** Tomography, X-ray computed, Cardiac-gated imaging techniques, Coronary artery disease, Diabetes mellitus, Myocardial infarction

## Abstract

**Objectives:**

To evaluate the prevalence of intra-myocardial fatty scars (IMFS) most likely indicating previous silent myocardial infarction (SMI), as detected on coronary artery calcium (CAC) computed tomography (CT) scans in diabetic patients without history of coronary heart disease (CHD).

**Methods:**

Diabetic patients screened for silent coronary insufficiency in a tertiary-care, university hospital between Jan-2015 and Dec-2016 were categorized according to their CAC score in two groups comprising 242 patients with CACS = 0 and 145 patients with CACS ≥ 300. CAC-CT scans were retrospectively evaluated for subendorcardial and transmural IMFS of the left ventricle. Adipose remodeling, patients’ characteristics, cardiovascular risk factors and metabolic profile were compared between groups.

**Results:**

Eighty-three (21%) patients with IMFS were identified, 55 (37.9%) in the group CACS ≥ 300 and 28 (11.6%) in the CACS = 0 (OR = 4.67; 95% CI = 2.78–7.84; *p* < 0.001). Total and average surface of IMFS and their number per patient were similar in both groups (*p* = 0.55; *p* = 0.29; *p* = 0.61, respectively). In the group CACS ≥ 300, patients with IMFS were older (*p* = 0.03) and had longer-lasting diabetes (*p* = 0.04). Patients with IMFS were older and had longer history of diabetes, reduced glomerular filtration rate, more coronary calcifications (all *p* < 0.05), and higher prevalence of carotid plaques (OR = 3.03; 95% CI = 1.43–6.39, *p* = 0.004). After correction for other variables, only a CACS ≥ 300 (OR = 5.12; 95% CI = 2.66–9.85; *p* < 0.001) was associated with an increased risk of having IMFS.

**Conclusions:**

In diabetic patients without known CHD, IMFSs were found in patients without coronary calcifications, although not as frequently as in patients with heavily calcified coronary arteries. It remains to be established if this marker translates in an upwards cardiovascular risk restratification especially in diabetic patients with CACS = 0.

**Clinical relevance statement:**

In diabetic patients without history of coronary heart disease, intramyocardial fatty scars, presumably of post-infarction origin, can be detected on coronary artery calcium CT scans more frequently, but not exclusively, if the coronary arteries are heavily calcified as compared to those without calcifications.

**Key Points:**

*• Intramyocardial fatty scars (IMFS), presumably of post-infarction origin, can be detected on coronary artery calcium (CAC) CT scans more frequently, but not exclusively, in diabetic patients with CACS ≥ 300 as compared to patients CACS = 0.*

*• Patients with IMFS were older and had longer history of diabetes, reduced glomerular filtration rate, and more coronary calcifications.*

*• Carotid plaques and CACS ≥ 300 were associated with an increased risk of having IMFS, about three and five folds respectively.*

**Supplementary Information:**

The online version contains supplementary material available at 10.1007/s00330-023-09940-2.

## Introduction

Complications from cardiovascular (CV) diseases account for the largest share of morbidity and mortality in patients with diabetes and present some specific characteristics in this population. First, both macrovascular disorders and microvascular dysfunction have been advocated [1]. Moreover, in patients with diabetes, coronary insufficiency is associated with a poorer prognosis but can remain undetected due to the absence of symptoms [2, 3]. While debate about the optimal strategy for the detection of silent coronary insufficiency is ongoing [4], guidelines prompt cardiovascular risk assessment in diabetic patients. Risk stratification aims to optimize multi-target medical treatment and to limit referral to further examination for the detection of silent ischemia and silent myocardial infarction (SMI) to the patients with the highest CV risk [5].

In this context, coronary artery calcium (CAC) burden, as assessed with unenhanced computed tomography (CT), is often employed in clinical practice since. Indeed, CAC has been proven superior to conventional cardiovascular risk factors for mid-term risk stratification in asymptomatic subjects with diabetes [6].

Although regarded as an efficient marker of coronary artery disease, CAC does not yield direct information about the presence of significant coronary stenosis nor silent ischemia. Nevertheless, unenhanced CT scans with cardiac synchronization can provide information about the myocardium. For instance, macroscopic intra-myocardial areas of adipose tissue can be detected due to the specific attenuation values of this tissue. It has been shown that adipose scar transformation of necrotic areas is a common phenomenon following myocardial infarction occurring in up to 84% of cases 3–5 years after the event [7, 8]. Several studies have demonstrated that CT can detect intramyocardial adipose tissue in the left ventricle, indicating areas of post infarction scars [9–11]. These fatty scars show a subendocardial and transmural localization typical of infarction physiopathology. Therefore, routinely performed CAC-CTs could be used to detect intramyocardial fatty scars (IMFS) resulting from unrecognized SMI. SMI are important to detect as they are an established cardiovascular risk marker associated with further cardiac events and poor prognosis in patients with diabetes [2, 12].

Hence, the aim of our study was to evaluate the prevalence of IMFS in diabetic patients without history of coronary heart disease (CHD), assessed by unenhanced CAC-CT scans, and to explore its association with CAC score (CACS), patients’ characteristics and metabolic profile. In this study, we focused the analysis on two clinically distinct groups of patients, representative of the two extremes of the spectrum of CV risk as identified with CAC: those with low (CACS = 0) and those with very high (CACS ≥ 300) CV risk.

## Materials and methods

### Patients’ population

In the present study, we retrospectively evaluated CAC-CT scans and other clinical characteristics of a sub-group of patients of the DISCO cohort.

The DISCO cohort [13], a retrospective descriptive monocentric cohort, was designed in the Diabetology Department of the Cardiovascular Hospital Louis Pradel, in Lyon (France). Seven hundred thirty-two diabetic patients  > 40 years old without history of CHD who had a CAC assessment between 01-Jan-2015 and 31-Dec-2016 were systematically included. CAC-CT was performed to identify very high-risk patients that would undergo further examinations for the detection of silent myocardial ischemia (as recommended by guidelines at the time of the study) [14]. Data regarding several biographic, biological and lifestyle parameters were collected in electronic medical records, with a high completeness (98%). This data collection was performed after agreement of the local ethics committee (Hospices Civils de Lyon, N°19-111). The database was declared to the national data protection committee (Commission nationale de l'informatique et des libertés, N°19-234) and all the patients received an information notice about the study in order to collect their possible refusal of participation, in compliance with the legislation in place at the time of the study (French bioethics law Jardé).

**Fig. 1:**
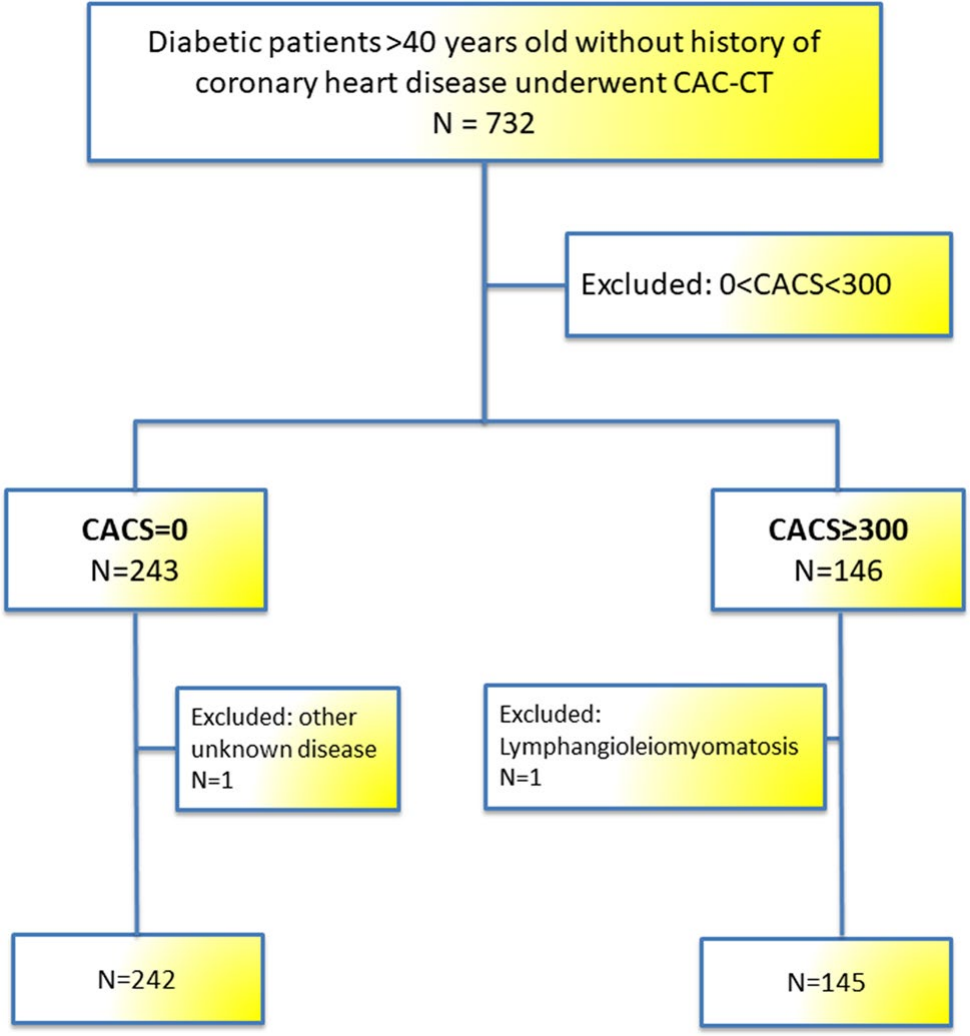
Flow chart of the study

Out of the DISCO cohort (*n* = 732), patients were included in the present sub-study based on the Agatston score. Only those with a CACS = 0 AU and those with a CACS ≥ 300 AU were further evaluated. These two groups correspond to a very low CV risk level and to a high CV risk, respectively [15] (Fig. 1). Hence, these two groups are two clinically distinct populations implying different management (primary vs secondary prevention).

### CT scan and Agatston score calculation

CT scans were performed according to routine recommended clinical protocols on a commercial CT scanner (Brilliance 64, Philips). Acquisition and reconstruction parameters are summarized in Supplemental Material-Table 1.

Agatston score was calculated during clinical routine by the radiologist in charge of the shift with a dedicated commercially available software (HeartBeat-CS, IntelliSpace Portal, Philips). Centiles were calculated based on the Multi-Ethnic Study of Atherosclerosis (MESA) database.

### Image analysis

CT scans of included patients were retrospectively analyzed, blinded to CAC score results, in consensus by two experienced observers in cardiovascular imaging.

Exams were reviewed on the PACS for the presence of intramyocardial fat. If the scan was deemed positive or doubtful, the images were exported to a commercial multimodality viewer (IntelliSpace Portal, Philips) for further analysis.

Intramyocardial fat was defined as the presence of a hypodense area inside the myocardium of the left ventricle with an average density ≤ 10 HU [9, 10]. The localization of each IMFS was defined according to the 17 segments AHA classification. In addition, its distribution inside the myocardial wall was noted as subendocardial, transmural (> 50% of the myocardial thickness) or located in the papillary muscle. Due to the difficulty in distinguishing the myocardium of the interventricular septum and the apical segments from the ventricular cavity, some IMFSs in this restricted area could not be categorized with certainty regarding their localization in the myocardial wall. Therefore, they were labelled as “septal” or “apical”. Fat containing areas of the right ventricular wall, left ventricle trabeculae and those with a clear epicardial localization were not taken into account for the purpose of this study.

For each IMFS, the surface, density and standard deviation of the density were calculated on the multiplanar reconstruction where the deposit appeared the largest. For each patient with multiple IMFS, the cumulative and average surface of all lesions were calculated.

### Comparison to scintigraphy

For patients having IMFS, images of scintigraphy performed within 1 year of the CAC-CT were retrospectively evaluated blinded to the surface and the topography of the IMFS. Analysis was performed by two independent experienced observers; doubts were settled by a third experienced observer.

### Statistics

Statistical analysis was performed with SPSS (IBM, version 21) and R (R project, version 4.0.4).

The categorical variables are presented as frequencies and percentages. The *χ*^2^ test or Fisher’s test was employed to analyze differences in categorical variables. For continuous variables, normality was assessed with Shapiro-Wilk’s tests and QQ-plots. Normally distributed values were expressed as average ± standard deviation. Values that were not normally distributed were expressed as median and interquartile range. Interquartile range was indicated as the difference between Q3 and Q1. *T*-Student’s and Mann-Whitney’s tests were employed accordingly to the distribution to assess differences of continuous and ordinal variables, respectively.

Simple logistic regression was used to calculate odds ratios of having IMFS. Furthermore, multiple logistic regression was used to assess the relationship of having an IMFS with the other predictors. Potential predictive variables were identified with a three-step process. Firstly, the variables that were considered the most relevant from a clinical point of view were selected. Secondly, in pairs of highly correlated predictors, one variable was disregarded. One final model was created. Thirdly, an Akaike Information Criterion (AIC) backward selection procedure was performed. For the selected variables, the odds ratios have been computed, and the Wald test performed (significant when *p* < 0.05).

## Results

Three hundred eighty-nine diabetic patients were analyzed, 243 patients had a CACS = 0 AU and 146 patients had a CACS ≥ 300 AU. Two patients, one per group, were excluded from further analysis (Fig. 1): one had diffuse fat infiltrate of his left ventricle in addition to recurrent pancreatitis and azoospermia and is currently under investigation; the other had several fat deposits and lung cystic lesions and was diagnosed with lymphangioleiomyomatosis. A total of 387 patients, 242 patients with CACS = 0 (62.5%) and 145 patients with CACS ≥ 300 (37.5%), were included for the final analysis.

The main characteristics of patients of groups CACS = 0 and CACS ≥ 300 are presented in Table 1. Additional patients’ data can be found in Supplemental Material-Table 2.

**Table 1:** Patients’ characteristics in the groups CACS = 0 and CACS ≥ 300 and IMFS characteristics

Patients’ characteristics	CACS = 0		CACS ≥ 300		*p*
	N*	Value	N*	Value	
Age (years)	242	61.2 ± 9.8	148	69.9 ± 9.1	** < 0.001**
Sex (male)	242	104 (43%)	145	93 (64%)	** < 0.001**
BMI (kg/m^2^)	241	29.3 (8.0)	142	29.0 (7.2)	0.81
Diabetes					
Diabetes type 1/2/3	241	63 (26%)/160 (66%)/18 (8%)	144	30 (21%)/102 (71%)/12 (8%)	0.72
Time since diagnosis (years)	241	17.9 (14.5)	147	24.9 (15.0)	** < 0.001**
HbA1c (%)	241	8.1 (2.3)	142	8.2 (2.0)	0.8
CAC					
Age at CAC (years)	242	56.4 ± 9.8	147	65.3 ± 9.0	** < 0.001**
CACS value	242	0	148	777 (703.0)	NA
CAC centile	242	0	144	94 (14.5)	NA
Intramyocardial fatty scars					
Patients with IMFS		28 (12%)		55 (38%)	** < 0.001**
Total N of IMFS		34		75	** < 0.001**
*N* of IMFS per patient					0.61^†^
1		22		39	
2		6		13	
3		0		2	
4–6		0		1	
Cumulative IMFS					
Total surface (mm^2^)		26.2 (23.3)		24.5 (25.8)	0.55
Average HU		1.7 (23.7)		− 5.3 (22.9)	0.16
Average HU SD		19.2 (10.9)		24.5 (11.4)	0.53
Average IMFS per patient					
Surface (mm^2^)		24.1 (22.9)		21.1 (16.4)	0.29
Localization in the myocardial wall					
Subendocardial		7 (21.0%)		24 (32.0%)	0.47^‡^
Transmural		11 (32.0%)		21 (28.0%)	
Papillary muscle		0		1 (1.0%)	
Septal/apical		16 (47.0%)		29 (39.0%)	

Categorical data are reported as number (percentage). Ordinal data are reported as average ± standard deviation or median (interquartile range, expressed as Q3–Q1) depending on the distribution. *Number of subjects with available data for the specific variable. †Calculated based on two groups of patients with one lesion and more than one lesion respectively. ‡Calculated excluding the papillary lesion. Type 3 diabetes included secondary diabetes (post pancreatic surgery, hemochromatosis, post pancreatitis, pancreatic neoplasia) and monogenic diabetes

Values in bold indicate significant differences

### IMFS

**Fig. 2:**
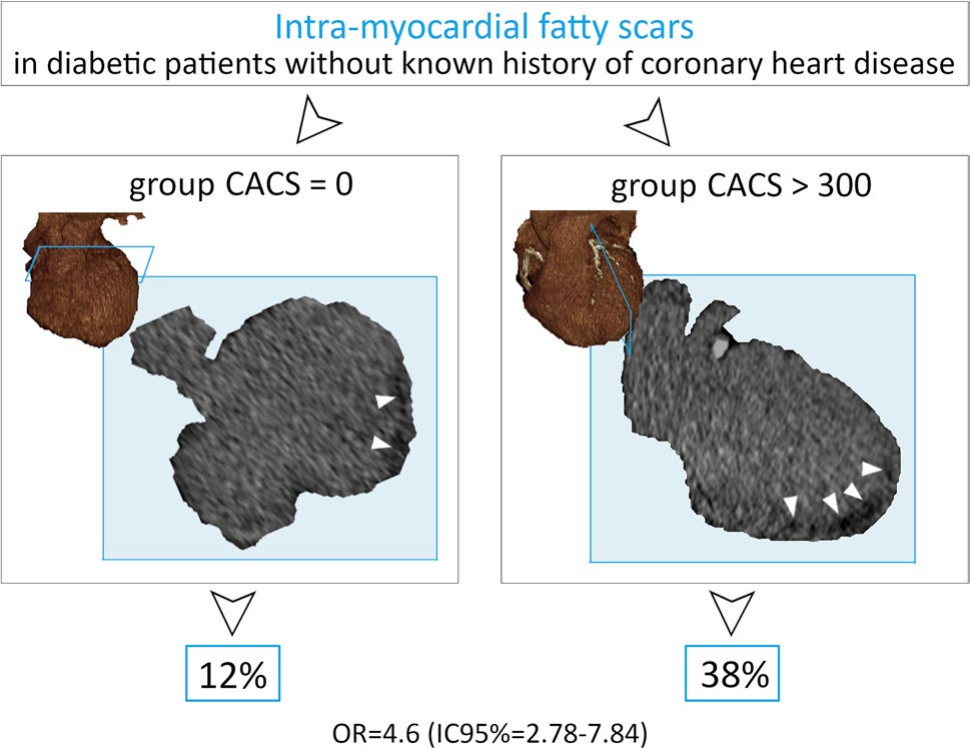
Different frequencies of intra-myocardial fatty scars (IMFS) in the CAC groups

**Fig. 3:**
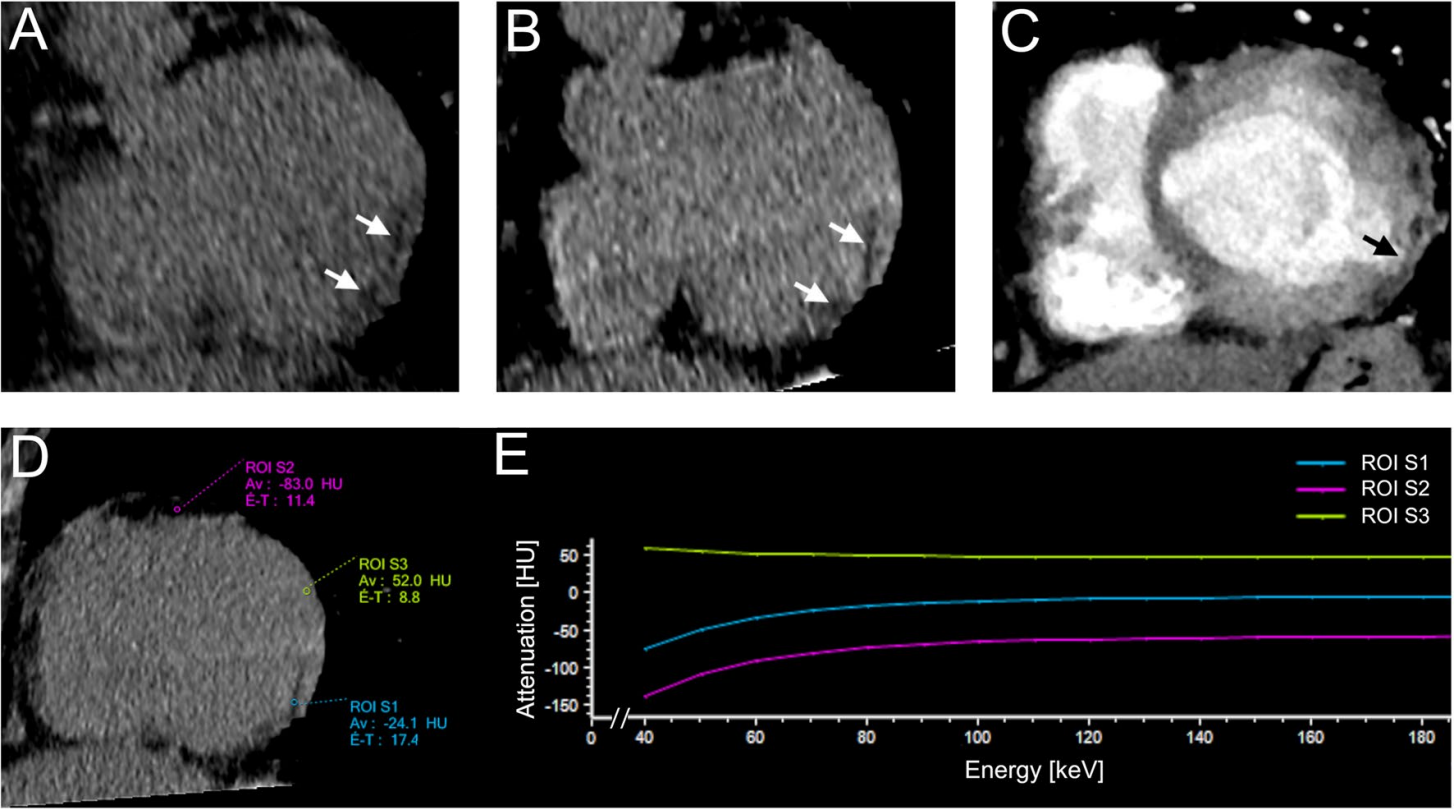
Examples of post-infarction IMFS from the CACS = 0 group. **A** A short axis multiplanar reconstruction of the first CAC-CT scan undergone by this patient showed a hypodense area with fat density contouring the subendocardium of the basal inferolateral wall of the left ventricle (arrows), indicating a previous SMI of this territory. **B** Another CAC-CT scan performed 3 years later confirmed the presence of this lesion (arrows). As this scan was performed on a dual-energy dual-layer CT scanner, the attenuation spectrum of this lesion could be analyzed and is shown in **D** and **E**. **C** About 1 year after the second CAC-CT, the same patient underwent a contrast-enhanced CT scan to rule out pulmonary embolism. Although the image quality is not satisfactory due to the lack of ECG-synchronization, the IMFS can be appreciated together with a thinning of the wall (arrow), another sign of post-infarction remodelling. **D** and **E** Voxel density analysis of the IMFS. **D** Three regions-of-interest (ROI) were placed in the myocardial lesion (ROI-S1), in the epicardial fat (ROI-S2) and in the myocardium (ROI-S3). **E** The ROI in the IMFS and in the epicardial fat both demonstrated attenuation values distribution (in Hounsfield unit (HU)) typical of fat (different from myocardial ROI-S3)

**Fig. 4:**
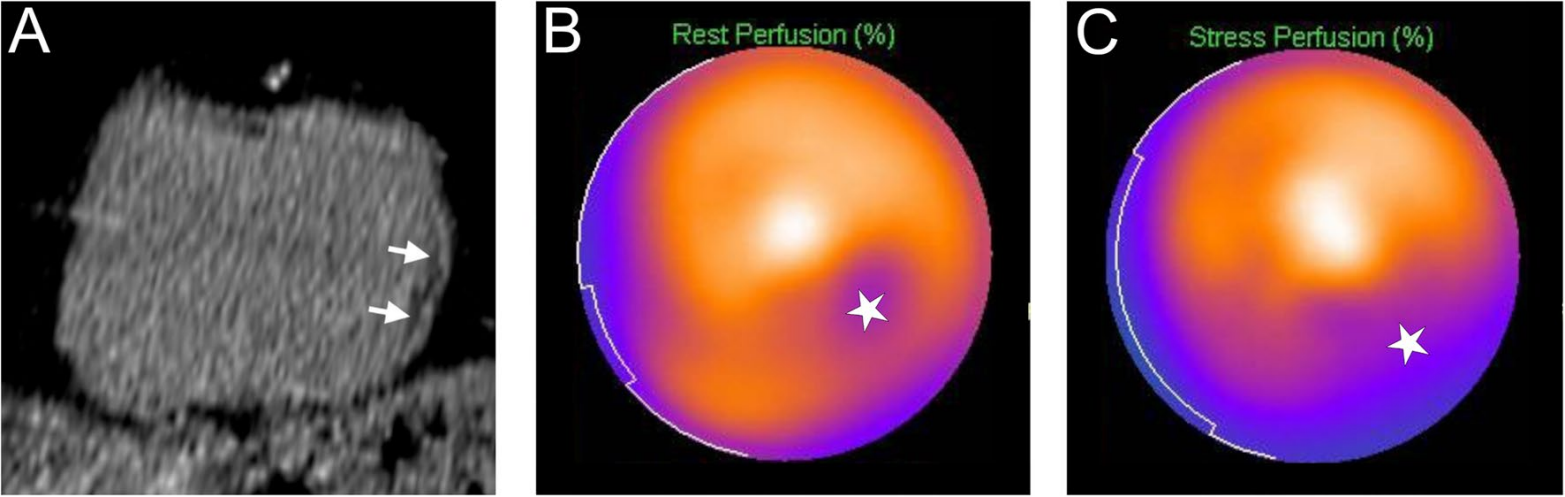
Examples of post-infarction IMFS from CACS ≥ 300 group. **A** CAC-CT short-axis reconstruction showing a subendocardial fat deposit of the mid-ventricular inferolateral wall of the left myocardium (arrows). A scintigraphy (**B** and **C**) realized 4 months after the CT confirmed the presence of a corresponding fixed perfusion defect (**B**, asterisk). In addition, the images acquired after stress (**C**) showed a larger area of ischemia (asterisk) surrounding the smaller necrotic zone

Overall 83 patients (21%) with fatty scars in the left ventricle myocardium were identified, of which 28 were in the group with a CACS = 0 (11.6%) and 55 in the group with a CACS ≥ 300 (37.6%), OR = 4.67 (95% CI = 2.78–7.84; *p* < 0.001) (Table 1 and Fig. 2). The difference was statistically significant even when all the IMFS in the apical segment 14 were excluded, leaving 17 lesions in the group CACs = 0 (7%) and 37 lesions in the group CACS ≥ 300 (25.5%; *p* ≤ 0.001). One example of IMFS per CAC group is shown in Figs. 3 and 4.

**Fig. 5:**
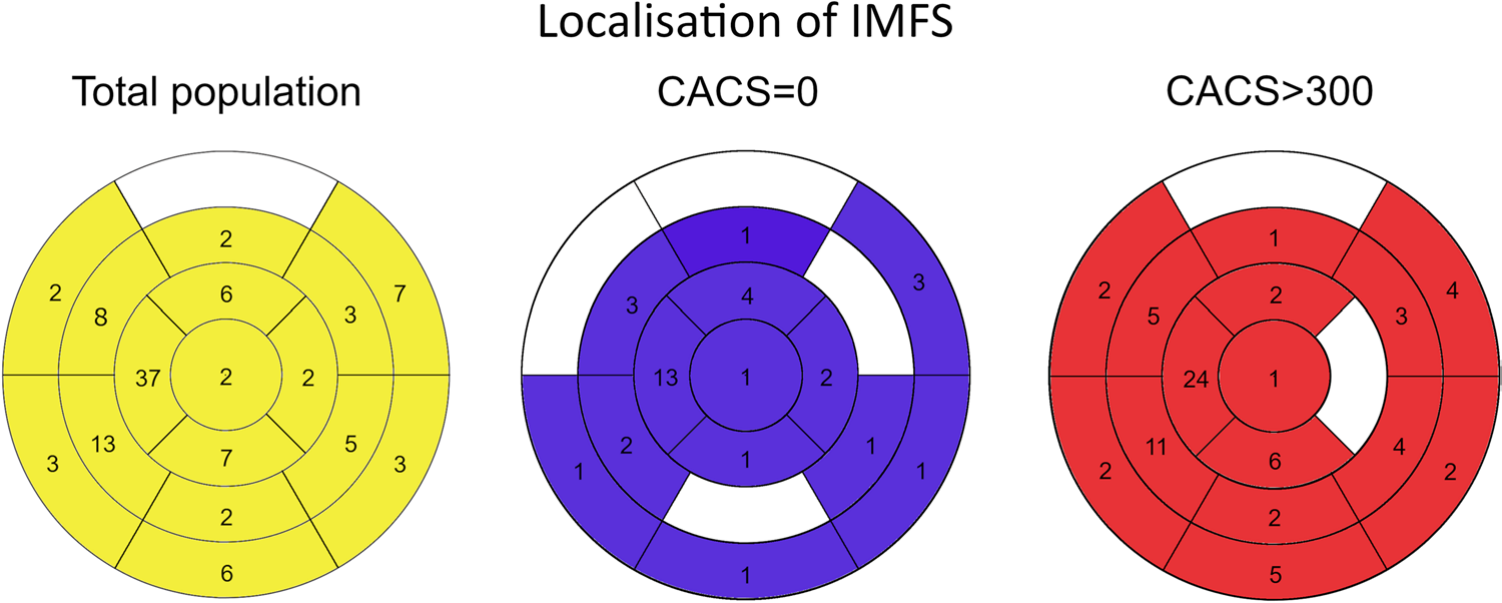
IMFS localization. The number of IMFS is reported for each of the conventional 16 segments of the left ventricle. IMFSs that were visible in two adjacent segments were counted twice, one for each segment

The number of IMFS, the cumulative surface and the average surface of fat lesions per patient who had an IMFS were similar in patients in the groups CACS = 0 and CACS ≥ 300 (Table 1). The distribution of the lesions in the 17 segments and the localization in the ventricular wall are reported in Table 1 and Fig. 5 respectively. Overall, a similar prevalence of IMFS was found in the sudendocardial and the transmural localization (Table 1).

### Comparison of demographics and metabolic profile between patients without and with IMFS per CACS group

Data for patients without and with IMFS split in the two CACS groups are presented in Table 2.

**Table 2:** Characteristics of patients without and with IMFS in the groups CACS = 0 and CACS ≥ 300

	CACS = 0					CACS ≥ 300				IMFS (CACS = 0 vs CACS ≥ 300)	
	No IMFS		IMFS			No IMFS		IMFS			
	N*	Value	N*	Value	*p*	N*	Value	N*	Value	*p*	*p*
Sex (male)	214	92 (43%)	28	12 (43%)	1	93	57 (63%)	53	36 (66%)	0.86	0.06
BMI (kg/m^2^)	213	29.3 (8.3)	28	29.7 (7.2)	0.91	87	29.4 (8.6)	55	28.3 (5.0)	0.42	0.50
Diabetes											
Diabetes type 1/2/3	213	55/141/17	28	8/19/1	0.915	89	16/66/7	55	14/36/5	0.598	0.556
		(26/66/8%)		(28/68/4%)			(18/74/8%)		(25/66/9%)		
Time since diagnosis (years)	213	16.9 (14.5)	28	19.4 (15.5)	0.92	89	23.9 (14.0)	55	25.9 (13.0)	0.2	**0.004**
HbA1c (%)	213	8.1 (2.4)	28	7.6 (1.7)	0.21	87	8.2 (2)	55	7.9 (2.2)	0.15	0.51
CAC											
Age at CAC (years)	214	56.3 ± 10.1	28	56.4 ± 7.4	0.82	90	64.1 ± 9.4	55	67.2 ± 9.4	0.052	** < 0.001**
CACS value		0		0		90	766.5 (658.5)	55	780 (783.0)	0.71	
CAC centile		0		0		87	94 (15.0)	55	93 (11.0)	0.27	
Risk factors											
Hypertension	213	123 (58%)	28	14 (50%)	0.54	87	65 (75%)	55	41 (75%)	1	**0.03**
DBP (mmHg)	213	72 (12.0)	28	75.5 (9.5)	**0.04**	87	72 (14.0)	55	75 (15)	0.87	0.18
SBP (mmHg)	211	127 (17.0)	28	130 (15.0)	0.24	87	130 (21.0)	55	130 (20.0)	0.37	0.92
Family history of MACE	212	18 (9%)	28	3 (11%)		79	14 (18.0%)	55	9 (16%)	1	0.74
Smoke	211		28		0.331	86		52		0.247	**0.006**
Active smoker		35 (16%)		2 (7%)			13 (15%)		7 (13%)		
Past smoker		41 (19%)		4 (14%)			26 (30%)		24 (44%)		
Laboratory data											
Triglycerides (mmol/L)	212	3.2 (2.5)	28	3.2 (2.5)	0.95	87	3.5 (2.6)	55	3.3 (2.2)	0.18	0.83
Total cholesterol (mmol/L)	210	4.8 (1.2)	28	4.9 (0.8)	0.89	86	4.4 (1.7)	55	4.3 (1.2)	0.45	**0.048**
HDL (mmol/L)	211	1.1 (0.4)	28	1.2 (0.4)	0.40	87	1.1 (0.5)	55	1.0 (0.4)	0.93	0.37
LDL (mmol/L)	211	2.9 ± 1.0	28	2.9 ± 0.8	0.81	87	2.6 ± 0.9	55	2.5 ± 0.9	0.63	0.07
Creatinine (mmol/L)	212	66 (23.0)	28	70.5 (21.3)	0.28	87	75 (31.0)	55	74 (31.0)	0.83	0.79
GFR (mL/min/1.73 m^2^)	212	99 (21.8)	28	98 (22.3)	0.31	87	90 (34.0)	55	83 (25.0)	0.29	**0.002**
Associated pathology											
Proteinuria	213		28		0.7	87		55		0.46	0.37
Microalbuminuria		58 (27%)		6 (21%)			33 (38%)		20 (36%)		
Macroalbuminuria		14 (7%)		1 (4%)			10 (12%)		3 (6%)		
Retinopathy	210		28		0.82	87		55		0.19	0.15
Minor		48 (23%)		6 (21%)			27 (31%)		9 (16%)		
Moderate		10 (5%)		1 (4%)			6 (7%)		5 (9%)		
Severe		21 (10%)		1 (4%)			19 (22%)		11 (20%)		
Autonomic neuropathy	212	11 (5%)	28	1 (4%)	1	87	6 (7%)	55	5 (9%)	0.75	0.66
Carotid plaque	193	112 (58%)	18	10 (56%)	1	82	73 (89%)	47	46 (98%)	0.09	** < 0.001**
Stroke	213	3 (1%)	28	0	1	87	7 (8%)	55	2 (4%)	0.48	0.55
Hepatic status	211		28		0.34	90		53		0.79	0.45
NAFLD		71 (70%)		11 (85%)			31 (72%)		18 (69%)		
NASH-cirrhosis-HCC		31 (30%)		2 (15%)			12 (28%)		8 (31%)		

Categorical data are reported as number (percentage). Ordinal data are reported as average ± standard deviation or median (interquartile range, expressed as Q3–Q1) depending on the distribution. *Number of subjects with available data for the specific variable

Values in bold indicate significant differences

*CAC*, coronary artery calcifications; *DBP*, diastolic blood pressure; *GFR*, glomerular filtration rate; *HCC*, hepatocellular carcinoma; *HDL*, high-density lipoproteins; *LDL*, low-density lipoproteins; *NAFLD*, non-alcoholic fatty liver disease; *NASH*, non-alcoholic steatohepatitis; *SBP*, systolic blood pressure

In the group with CACS = 0, the only significant differences were a mild increase in diastolic blood pressure in the subgroup with IMFS (75.5 vs 72 mmHg; *p* = 0.04) and more patients treated with calcium channel blockers in the subgroup without IMFS (17.4% vs 0; *p* = 0.02).

In the group with CACS ≥ 300, patients with IMFS had undergone their CAC-CT assessment at a later age (67.2 vs 64.1 years; *p* = 0.04).

Differences between patients with IMFS that had a CACS = 0 and a CACS ≥ 300 are shown in the last column of Table 2. Among patients with IMFS, patients with CACS ≥ 300 were 11 years older at the time of the CAC assessment (67.2 ± 9.4 vs 56.43 ± 7.39 years), had a 7 years longer duration of diabetes (25.9 (13.0) vs 19.4 (15.5) years) and a 15 mL/min/1.73 m^2^ reduced glomerular filtration rate (GFR) (83 (25.0) vs 98 (22.3) 15 mL/min/1.73 m^2^) as compared to patients with CACS = 0 (all *p* < 0.05). The same subgroup of patients also had more frequently hypertension (75% vs 50%; *p* = 0.03) and was more likely to include present or past smokers (45% vs 21%; *p* = 0.006). Furthermore, among patients with IMFS, patients with CACS ≥ 300 had more frequently (almost 9 patients out of 10 vs less than one-third) carotid plaques at ultrasound (*p* < 0.001).

### Comparison of demographics and metabolic profile between patients without and with IMFS

Data of patients without and with IMFS is presented in Table 3 and additional data about medications in these groups in Supplemental Material—Table 3.

**Table 3:** Characteristics of patients without and with IMFS

	Without IMFS	IMFS	*p*	Odds	CI 95%	Sig
	Value	Value				
Sex (male)	149 (49%)	48 (58%)	0.17	1.43	0.87–2.33	0.16
BMI (kg/m^2^)	29.4 (8.2)	28.7 (5.0)	0.48	0.98	0.95–1.02	0.44
Diabetes						
Diabetes type 1/2/3	71/207/24	22/55/7	0.89			0.91
	(23/69/8%)	(27/66/8%)				
Time since diagnosis (years)	18.9 (15.0)	21.9 (14.0)	**0.008**	1.01	1–1.03	0.13
HbA1c (%)	8.2 (2.4)	7.8 (2.0)	0.09	0.85	0.74–0.99	**0.034**
CAC						
Age at CAC (years)	58.6 ± 10.5	63.5 ± 9.3	**0.001**	1.05	1.02–1.08	** < 0.001**
CACS value	0 (413.0)	496 (901.0)	** < 0.001**			
CAC centile	0 (75.0)	86 (95.0)	** < 0.001**			
Risk factors						
Hypertension	188 (63%)	55 (66%)	0.61	1.17	0.70–1.95	0.55
DBP (mmHg)	72 (13.0)	75 (10.0)	0.14	1.01	0.99–1.04	0.32
SBP (mmHg)	129 (15.0)	130 (19.0)	0.35	1.01	0.99–1.02	0.40
Family history of MACE	32 (11%)	12 (15%)	0.44	1.39	0.67–2.79	0.39
Smoke			0.09			0.85
Active smoker	48 (16%)	9 (11%)				
Past smoker	67 (23%)	28 (34%)				
Laboratory data						
Triglycerides (mmol/L)	3.3 (2.6)	3.3 (2.2)	0.42	0.92	0.77–1.10	0.36
Total cholesterol (mmol/L)	4.8 ± 1.6	4.6 ± 1.0	0.33	0.66	0.38–1.13	0.13
HDL (mmol/L)	1.1 (0.4)	1.1 (0.4)	0.85	1.04	0.19–5.79	0.97
LDL (mmol/L)	2.8 ± 1.0	2.7 ± 0.9	0.36	0.66	0.33–1.3	0.23
Creatinine (mmol/L)	68 (25.0)	72 (29.0)	**0.04**	1.01	1–1.02	0.13
GFR (mL/min/1.73 m^2^)	95 (25.0)	86 (26.0)	**0.002**	0.98	0.97–0.99	**0.006**
Associated pathology						
Proteinuria			0.7			0.62
Microalbuminuria	91 (30%)	26 (31%)				
Macroalbuminuria	24 (8%)	4 (5%)				
Retinopathy			0.53			0.57
Minor	75 (25%)	15 (18%)				
Moderate	16 (5%)	6 (7%)				
Severe	40 (14%)	12 (15%)				
Autonomic neuropathy	17 (6%)	6 (7%)	0.60	1.29	0.49–3.39	0.6
Carotid plaque	185 (67%)	56 (86%)	**0.002**	3.03	1.43–6.39	**0.004**
Stroke	10 (3%)	2 (3%)	1	0.67	0.15–3.33	0.67

Categorical data are reported as number (percentage). Ordinal data are reported as average ± standard deviation or median (interquartile range, expressed as Q3–Q1) depending on the distribution. *Number of subjects with available data for the specific variable

Values in bold indicate significant differences

*CAC*, coronary artery calcifications; *DBP*, diastolic blood pressure; *GFR*, glomerular filtration rate; *HCC*, hepatocellular carcinoma; *HDL*, high-density lipoproteins; *LDL*, low-density lipoproteins; *NAFLD*, non-alcoholic fatty liver disease; *NASH*, non-alcoholic steatohepatitis; *SBP*, systolic blood pressure

Patients with IMFS were about 4 years older at CAC assessment (63.5 ± 9.3 vs 58.6 ± 10.5 years; *p* = 0.001), had more coronary calcifications (CACS increased of 496 AU and 86 centiles (*p* < 0.001)), had a 3 years longer duration of diabetes (21.9 (14.0) vs 18.9 (15.0) years; *p* = 0.008) and had a 9 mL/min reduced renal function as compared to patients without IMFS (86 (26.0) vs 95 (25.0) mL/min/1.73 m; *p* = 0.002).

Patients using statins had a 1.75 (95% IC = 1.07–2.88; *p* = 0.03) higher risk and those showing carotid plaques at ultrasound had a risk increase of 3.03 folds (95% IC = 1.43–6.39; *p* = 0.004) of having IMFS (Table 3).

Multiple logistic regression (Table 4) showed a negative association between IMFS and levels of glycated haemoglobin (OR = 0.80; 95% IC = 0.66–0.96; *p* = 0.019) and confirmed a strong positive association with CACS ≥ 300 (OR = 5.12; 95% IC = 2.66–9.85; *p* < 0.001). Age at CAC was among the predictors attained in the reduced model but was not significant.

**Table 4:** Multiple logistic regression

Odds ratio estimates				
Effect	Point estimate	95% Wald confidence limits		*p*-value
Age at CAC	1.03	0.993	1.059	0.120
HbA1c	0.80	0.668	0.966	0.020
CACS 300 vs 0	5.12	2.664	9.853	< 0.001

Variables: sex, age at CAC, BMI, hypertension, total cholesterol, glycated haemoglobin, smoke, presence of carotid plaques, CAC group, creatinine

### Comparison between IMFS and results of scintigraphy

Eighty-one patients (20% of the total), of which only one in the CACS = 0 group, underwent a scintigraphy within 1 year from the CAC assessment. Twenty-nine exams were performed in patients with IMFS, out of which 13 were classified as pathological. Among these, 8 were deemed positive for necrosis with or without associated ischemia, 2 doubtful or with small lesions and 3 with ischemia without necrosis. Among these 8 patients, 6 had at least one SMI fat deposit on the CAC-CT and 2 had two. In all the 8 cases, the localization of at least one of the lesions visible on the scintigraphy corresponded to that of at least one of the IMFS.

## Discussion

Our results show that unenhanced CT scans performed to quantify CAC enabled the identification of intramyocardial fatty scars of presumed myocardial infarction origin in one out five diabetic patients without history of CHD. These IMFSs were more frequent in patients with high CACS as compared to patients with CACS = 0. Older age at CAC assessment, higher CAC centile, reduced GFR and the presence of carotid plaques were associated with an increased probability of having fatty scar of previous SMI, up to 3 folds for carotid plaques. After adjustment for other factors, only CACS ≥ 300 was associated with an increased prevalence of IMFS.

In patients with diabetes, SMI as assessed by electrocardiogram (ECG), scintigraphy or magnetic resonance imaging (MRI) is associated with an increased rate of future events. Indeed, the incidence of major cardiac events and death is significantly increased in asymptomatic diabetic patients with SMI [12, 16–19]. Interestingly, the risk of future events after SMI is similar to that encountered in patients with a history of symptomatic MI [18]. Consequently, it is important to identify SMI. The possibility to detect fatty scars of MI on a non-invasive and affordable imaging examination (such as unenhanced CAC-CT) may provide a unique opportunity. The fact that CAC-CT is already integrated in the management of diabetic patients certainly adds to the interest.

Fat tissue deposition in areas of necrosis is a well-documented occurrence after myocardial infarction in ovine and rabbit models as well as in humans [7, 20–22]. This appears to be a continuous process, visible 3–5 years after the event and then progressing to replace up to 50% of the extent of the necrotic area in the 15 years after [8, 11]. The extent of the fatty scar correlates not only with the size of the infarction but also with segmental thinning and reduced contractility of the myocardium as well as global left ventricle function [23, 24]. Moreover, the presence of fatty scars is associated with altered electrophysiological properties of the left ventricle wall and with recurrent ventricular tachycardia [11, 22].

These fatty scars can be found within, or interspersed with, fibrous tissue replacing necrosed myocardium. As such, subendocardial and transmural fatty scars are a distinct sign of previous infarction and not of ischemia as the latter does not result in fibrotic and adipose replacement. Their specific localization in the myocardium, subendocardial with possible transmural extension, reflects the physiopathology of infarction [11, 21]. As a matter of fact, left ventricle fat lesions can sometimes be observed in other rare pathologies with non-specific localization [25]. In addition, myocardial adipose tissue inclusions have been described in 0.2–3% [8, 26] of patients without any known disease, mainly in the trabeculae and in the basal segments [25, 27].

Diabetic patients have lipid metabolism dysregulation leading to myocardial steatosis that consists of triglycerides accumulation in myocardiocytes of the entire myocardium [5]. Therefore, myocardial steatosis cannot be visualized on CT scans.

Henceforth, subendocardial and transmural fatty lesions are most likely post-infarction scars.

Conveniently, post-infarction fatty scars are detectable with non-invasive imaging technique such as CT and MRI [9, 10]. Although a review speculated that fat depositions could be detected on CAC-CT [28], no report on this topic is available in a large cohort of patients with diabetes.

Our findings show that these IMFSs are found in 38% of asymptomatic patients with diabetes and high calcium score. These results are in line with previous studies reporting a prevalence of 10–37% of SMI in diabetic patients with several cardiovascular risk factors [2]. Likewise, in a cohort of patients with similar characteristics, MRI showed late gadolinium enhancement indicating SMI scars in 28% of patients [16].

We also found a prevalence of 11.6% of IMFSs in patients without known CHD and with no coronary calcifications. This percentage appears to be higher than the reported 3.8% of asymptomatic patients without known CHD showing Q waves in the Femantle study [29], possibly due to the lack of sensitivity of ECG. In agreement with our findings, Elliot et al [30] recently found signs of SMI on late enhancement MRI in 13% of asymptomatic, average risk diabetic patients.

In current clinical routine, diabetic patients with CACS = 0 are considered at low CV risk and treated accordingly. Nevertheless, since the IMFSs we described are most likely signs of previous infarction, cardiovascular prevention should probably be intensified in the patients with a CACS = 0 and IMFS. Prospective controlled studies will have to ascertain if the subgroup of patients with a CACS = 0 and SMI scars has an unexpectedly high frequency of CV complications.

Interestingly, we found similar cumulative and average surface of IMFSs in both groups of CAC. Whether any (and which) conclusions could be drawn from these findings remains unclear. On one hand, the presence of relatively frequent but small IMFSs in patients CACS = 0 could lure one to believe that they represent infarction of microvascular origin. On the other hand, the finding of IMFSs with similar characteristics in patients with known epicardial coronary atherosclerosis, as highlighted by the high CAC, undermines this assumption. In any case, the small dimensions of the IMFSs in our study are concordant with results from Amier et al [31] and Elliot et al [30]. Their analysis of MR examinations demonstrated SMI areas of late enhancement corresponding to an average mass of 5 g in the general population and to 5.1% (6–4.2%) of the left ventricle myocardium in diabetic patients, respectively.

In our cohort, patients with fatty scars of SMI were older, presented higher CAC scores and centiles, and had longer duration of diabetes and reduced renal function. These results are in agreement with previous works on SMI in the diabetic population [2, 18]. Furthermore, we found that one of the predictors for SMI was the detection of carotid plaques with ultrasound, a well-known, good predictor of cardiovascular complications, atherosclerosis being a systemic disease [32, 33]. On the other hand, the unexpected finding of similar levels of albuminuria and LDL cholesterol in the two groups of our study may be due to the high incidence of statin and ACE inhibitors prescription.

In our study, in 6 out of 8 cases where scintigraphy exams indicated the presences of a necrotic area in the myocardium, CT scans revealed a corresponding IMFS. Failure of scintigraphy to identify the remaining cases of previous SMI is not surprising. In fact, it can be ascribed, in primis, to the lower spatial resolution of this technique as compared to CT or MRI [34]. In addition, scintigraphy has demonstrated a lower accuracy for the detection of subendocardial lesions as compared to MRI in animal and human studies [35–38]. Both these elements have surely contributed to the fact that, in our study, some small necrotic areas (visible on CT as IMFS) were not detected in scintigraphy.

Two important limitations in this study need to be addressed. The first one is that, given the absence of an appropriate comparison method such as MRI, we cannot be absolutely certain that all the fatty areas we described were scars of infarction. However, our criteria for the identification of IMFSs are based on a rather vast literature and include morphology and location of the scars. The strict adhesion to these criteria substantiates our findings. The second limitation is that prospective data to validate IMFS as a CV risk marker are needed in order to support any modulation in the clinical management of these patients.

In conclusion, IMFSs likely indicating previous unrecognized SMI in diabetic patients without history of coronary heart disease are more frequently, but not exclusively, found in patients with CACS ≥ 300. Therefore, longitudinal studies should be conducted in order to establish if the presence of these IMFS should prompt a modification of the CV risk stratification.

### Supplementary Information

Below is the link to the electronic supplementary material.

Supplementary file1 (PDF 183 KB)

## Abbreviations

AU: Agatston unit
CAC: Coronary artery calcium
CACS: Coronary artery calcium score
CHD: Coronary heart disease
CT: Computed tomography
CV: Cardiovascular
ECG: Electrocardiogram
GFR: Glomerular filtration rate
IMFS: Intra-myocardial fatty scar
MACE: Major adverse cardiac events
MESA: Multi-ethnic study of atherosclerosis
MI: Myocardial infarction
MRI: Magnetic resonance imaging
SMI: Silent myocardial infarction

## Declarations

### Guarantor

The scientific guarantor of this publication is Pr Philippe Moulin.

### Statistics and biometry

Fabio Rapallo kindly provided statistical advice for this manuscript.

### Study subjects or cohorts overlap

The same cohort of patients has already been the object of a previous analysis: “Higher prevalence of incidental findings identified upon coronary calcium score assessment in type 2 and type 3 diabetes versus type 1 diabetes” by M Gaudillière, C Marsot, L Balaire, L Groisne, M Moret, S Villar-Fimbel, P Douek, P Moulin and S Charrière, https://doi.org/10.1371/journal.pone.0251693. However, the previous study examined completely different variables than the ones we have focused on in the present study. Therefore, there is no overlap with the present study.

### Methodology

• retrospective

• cross sectional study

• performed at one institution
