# Supplementary material for: Silent myocardial infarction fatty scars detected by coronary calcium score CT scan in diabetic patients without history of coronary heart disease
Source: Eur Radiol. 2023 Aug 2;34(1):214–25. doi: 10.1007/s00330-023-09940-2 (PMC10791785; doi:10.1007/s00330-023-09940-2)
Supplement: Supplementary file 1 — Supplementary file1 (PDF 183 KB) [file 330_2023_9940_MOESM1_ESM.pdf]

Online-only supplemental material

Supplemental Material- Table 1 – CT acquisition and reconstruction parameters

**CT parameters**	
**Acquisition**	
Type of acquisition	Prospectively triggered
Cardiac phase	Phase 75%
Contrast	Unenhanced
Collimation	32x0.625mm
kV	120 kV
mA	232
Rotation time	0.27
**Reconstruction**	
FOV	Adapted to the heart of the patient
Matrix	512
Slice thickness [mm]	2.5
Kernel	Soft (CB)

Supplemental Material- Table 2 – Additional patients' characteristics in the groups CACS=0 and CACS≥300

	CACS=0		CACS≥300		
	**N***	**value**	**N***	**value**	**p**
**Risk factors**					
Hypertension	241	137 (57%)	142	106 (75%)	**<0.001**
DBP (mmHg)	241	73 (11.0)	142	73.5 (14.0)	0.93
SBP (mmHg)	241	127 (15.5)	142	130 (20.0)	**<0.001**
Familiarity	240	21 (9%)	134	23 (17%)	**0.019**
Smoke	239		140		**0.001**
Active smoker		37 (16%)		20 (14%)	
Past smoker		45 (19%)		50 (36%)	
**Laboratory data**					
Triglycerides (mmol/L)	240	3.2 (2.5)	142	3.4 (2.3)	0.5
Total cholesterol (mmol/L)	237	4.8 (1.7)	141	4.4 (1.5)	**0.02**
HDL (mmol/L)	239	1.1 (0.4)	142	1.1 (0.5)	0.22
LDL (mmol/L)	239	2.9±0.9	142	2.6±1.3	**<0.001**
Creatinine (mmol/L)	240	67 (23.0)	142	75 (30.5)	**<0.001**
GFR (mL/min/1.73m^2)	240	98 (22.0)	142	85 (27.0)	**<0.001**
**Associated pathology**					
Proteinuria	241		142		**0.028**
Microalbuminuria		64 (27%)		53 (37%)	
Macroalbuminuria		15 (6%)		13 (9%)	
Retinopathy	238		142		**0.001**
Minor		54 (23%)		36 (25%)	
Moderate		11 (5%)		11 (8%)	
Severe		22 (9%)		30 (21%)	
Autonomic neuropathy	240	12 (5%)	142	11 (8%)	0.19
Carotid plaque	211	122 (58%)	129	119 (92%)	**<0.001**
Stroke	241	3 (1%)	142	9 (3%)	**0.008**
Lower limb arteriopathy	26	18 (69%)	42	39 (93%)	**0.014**

Hepatic status	115		69		0.55
NAFLD		82 (71%)		49 (71%)	
NASH-cirrhosis-HCC		33 (29%)		20 (29%)	
**Medication**					
Insulin	242	121 (50%)	145	90 (62%)	**0.03**
Statins	241	101 (42%)	142	93 (66%)	**<0.001**
Fibrate	241	3 (1%)	142	6 (2%)	0.83
Ezetimibe	241	14 (6%)	142	6 (4%)	0.64
Aspirin	241	43 (18%)	142	54 (38%)	**<0.001**
Other anti-platelet drugs	241	5 (2%)	142	14 (4%)	**0.001**
ACE-inhibitors	241	128 (53%)	142	98 (69%)	**0.003**
Calcium channel blockers	241	37 (15%)	142	41 (29%)	**0.002**
Beta-blockers	241	30 (12%)	142	23 (16%)	0.36
Metformin	241	151 (63%)	142	93 (66%)	0.59
Sulfonylureas	241	83 (34%)	142	47 (33%)	0.82
Gliptins	241	56 (23%)	142	29 (20%)	0.61
GLP-1ag	241	34 (14%)	142	22 (16%)	0.77

Categorical data are reported as: number (percentage). Ordinal data are reported as: average±standard deviation or median (interquartile range, expressed as Q3-Q1) depending on the distribution. *number of subjects with available data for the specific variable. CAC: coronary artery calcifications; DBP: diastolic blood pressure; GFR: glomerular filtration rate; HCC: hepatocellular carcinoma; HDL: high-density lipoproteins; LDL: low-density lipoproteins; NAFLD: non-alcoholic fatty liver disease; NASH: non-alcoholic steatohepatitis; SBP: systolic blood pressure.

Supplemental Material- Table 3 – Medications of patients without and with IMFS

	Without IMFS		IMFS					
	**N***	**Value**	**N***	**Value**	**p**	**Odds**	**CI 95%**	**Sig**
**Medication**								
Insulin	300	164 (54%)	83	47 (57%)	0.65	1.11	0.68-1.82	0.66
Statins	300	143 (48%)	83	51 (61%)	**0.035**	1.75	1.07-2.88	**0.027**
Fibrate	300	5 (2%)	83	4 (5%)	0.11	2.99	0.78-11.39	0.11
Ezetimibe	300	17 (6%)	83	3 (4%)	0.59	0.62	0.18-2.18	0.46
Aspirin	300	76 (25%)	83	21 (25%)	1	0.99	0.57-1.75	0.99
Other anti-platelet drugs	300	15 (2%)	83	4 (5%)	1	0.96	0.31-2.98	0.95
ACE-inhibitors	300	176 (59%)	83	50 (60%)	0.9	1.07	0.65-1.75	0.8
Calcium channel blockers	300	63 (21%)	83	15 (18%)	0.65	0.83	0.44-1.55	0.56
Beta-blockers	300	43 (14%)	83	10 (12%)	0.72	0.82	0.39-1.71	0.59
Metformin	300	190 (63%)	83	54 (65%)	0.79	1.08	0.65-1.79	0.77
Sulfonylureas	300	99 (33%)	83	31 (63%)	0.51	1.21	0.73-2.01	0.46
Gliptins	300	72 (24%)	83	13 (16%)	0.14	0.59	0.31-1.16	0.11
GLP-1ag	300	41 (13%)	83	15 (18%)	0.38	1.39	0.73-2.67	0.32

Categorical data are reported as: number (percentage). *number of subjects with available data for the specific variable
